# Acute Respiratory Distress Syndrome: An Unexpected Outcome of Suspected Viral Gastroenteritis

**DOI:** 10.7759/cureus.18539

**Published:** 2021-10-06

**Authors:** Oliver J Chiong, Michelle M Lu

**Affiliations:** 1 General Practice, Naval Hospital Camp Pendleton, Oceanside, USA

**Keywords:** military healthcare, septic shock, severe sepsis, gastrointestinal infection, enterovirus, myocarditis, acute respiratory distress syndrome [ards]

## Abstract

Acute respiratory distress syndrome (ARDS) is a life-threatening manifestation of diffuse inflammation damaging the lung pleura. Risk factors for development are numerous with most cases arising in those already hospitalized for critical illness. We describe a unique case of a healthy 20-year-old female developing myocarditis and severe ARDS while hospitalized for septic shock after initially presenting with gastroenteritis from a suspected Coxsackie B infection in the setting of an overseas military deployment. After two transfers via land and air, she reached a facility that delivered definitive care and survived. This case highlights how a common disease can develop into something far more deadly and how early recognition of ARDS risk factors can improve clinical decision-making at the time of admission.

## Introduction

Acute respiratory distress syndrome (ARDS) is the result of massive inflammation-causing diffuse alveolar damage leading to impaired gas exchange and resultant hypoxemic respiratory failure. ARDS is diagnosed using the Berlin criteria, which requires the onset of hypoxemic respiratory failure requiring ventilator support with evidence of bilateral infiltrates on chest x-ray within a week of a defined clinical injury such as pneumonia, aspiration, sepsis, major surgery, or trauma [1,2]. Based on the ratio of partial pressure of oxygen to fractional inspired oxygen (PaO_2_:FiO_2_), ARDS is further stratified to mild, moderate, and severe with the observed mortality being 27%, 32%, and 45%, respectively [1,2]. Pneumonia, sepsis, and aspiration account for as much as 85% of all ARDS cases [2]. Pulmonary sources of sepsis and non-pulmonary sources account for as much as 46% and 33%, respectively [3]. 

We describe the case of a healthy 20-year-old active-duty female as she initially presents with gastroenteritis and developed septic shock followed by myocarditis and severe ARDS while on a military deployment where medical capability is limited. With close coordination of military medical assets across geographic regions, she survived.

## Case presentation

A healthy 20-year-old active-duty female with no significant past medical history presented to our expeditionary medical clinic located on a remote military base in Kuwait for 10 hours of nausea, non-bloody, non-bilious vomiting; and non-bloody diarrhea after reportedly eating at a fast-food stand a day prior. She denied fever, chills, chest pain, dyspnea, or abdominal pain. She had tachycardia at 112 bpm with remaining vitals normal (BP: 116/72 mmHg, RR: 16, T: 99.6°F). She was well-appearing and in no significant distress. On exam, she had clear lungs, no rashes, abdominal tenderness, rebound, or guarding. We administered intravenous fluids with improvement in tachycardia to 100 bpm and she was discharged to quarters with supportive care.

Early next morning, the patient was escorted in when her roommates found her unconscious and covered with vomit and fecal matter. On assessment, she was awake but unresponsive. (Glasgow Coma Scale [GCS]: E4V1M4). Her vitals were blood pressure of 118/80 mmHg, heart rate of 136 bpm, temperature of 103.5°F, and respiratory rate of 20. Her fingerstick blood sugar was 134 mg/dL, and the rapid COVID-19 antigen test was negative. After initial fluid resuscitation, her vitals showed blood pressure was 65/35 mmHg, heart rate was 120, respiratory rate of 24, and oxygen saturation by pulse oximetry was 92% on room air. We administered norepinephrine, acetaminophen, empiric ciprofloxacin, and metronidazole for gastrointestinal infection. We then transferred her by ground ambulance to a nearby United States military hospital.

Upon arrival at the hospital, her blood pressure was in the 80s/40s mmHg with tachycardia decreased to 111 bpm, respiratory rate at 16, and her temperature was 99.6°F. A COVID-19 polymerase chain reaction (PCR), and urine drug screen were negative. Urinalysis showed trace leukocyte esterase with negative nitrites. Additional admission labs are shown in Table 1. A chest x-ray showed hypo-inflated lungs but no signs of acute cardiopulmonary processes. The patient’s mental status improved, and she could answer questions appropriately. She was admitted to the intensive care unit (ICU) with a diagnosis of sepsis secondary to a gastrointestinal infection. The ICU team continued her on metronidazole and added ceftriaxone for empiric urinary coverage.

**Table 1:** Labs as trended over initial hospital stay AST: Aspartate aminotransferase, ALT: Alanine aminotransferase, CK-MB: creatine kinase myocardial band

Lab	Admission	Day 1	Day 2	Reference Ranges
Sodium	132	130	132	137-145 mmol/L
Potassium	3.1	2.9	3.4	3.5-5.1 mmol/L
Chloride	104	108	101	98-107 mmol/L
Bicarbonate	17	21	28.0	22-30 mmol/L
Blood Urea Nitrogen	37	21	11	7-21 mg/dL
Creatinine	4	1.5	0.8	0.8-1.4 mg/dL
Glucose	157	130	181	72-113 mg/dL
Calcium	7.8	7.4	7.0	8.9-10.3 mg/dL
Total Protein	4.7	4.4	4.6	6.3-8.2 g/dL
Albumin	2.6	2.2	2.3	3.3-5.5 g/dL
AST	192	84	240	15-46 U/L
ALT	108	134	108	5-70 U/L
Alkaline Phosphatase	47	48	58	38-126 U/L
Total Bilirubin	1.6	1.2	1.0	0.8-1.4 mg/dL
Lactate	4.9	2.99	1.97	0.7-2.0 mmol/L
White Blood Cell	12.7	10.4	10.4	4.5-11 x 10^3^/µL
Hemoglobin	11.4	9.8	11.1	11.3-15.5 g/dL
Platelets	96	109	56	110-440 x 10^3^/µL
Myoglobin	>500	>500	370	0-107 ng/mL
CK-MB	21.0	80	35.6	1.4-4.3 ng/mL
Troponin I	0.23	30	20	0.0-0.4 ng/mL

**Figure 1:**
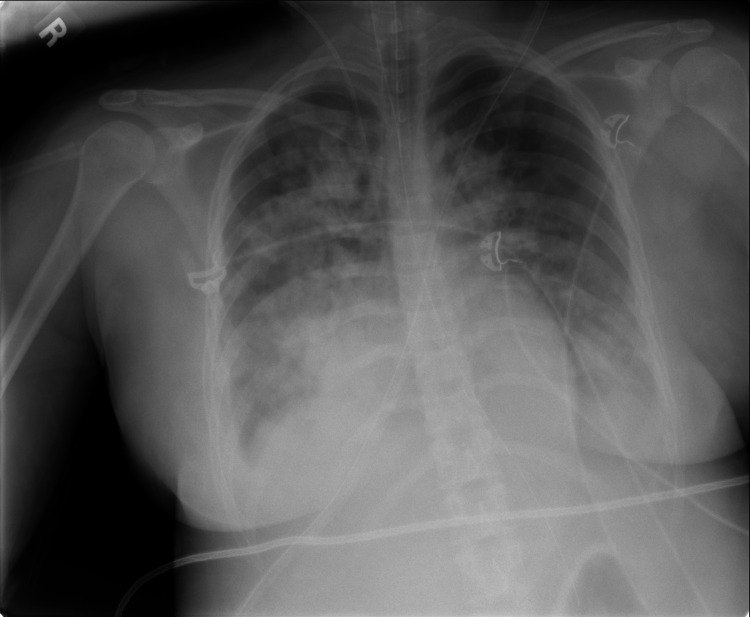
Chest x-ray showing bilateral infiltrates with right-side predominance

**Figure 2:**
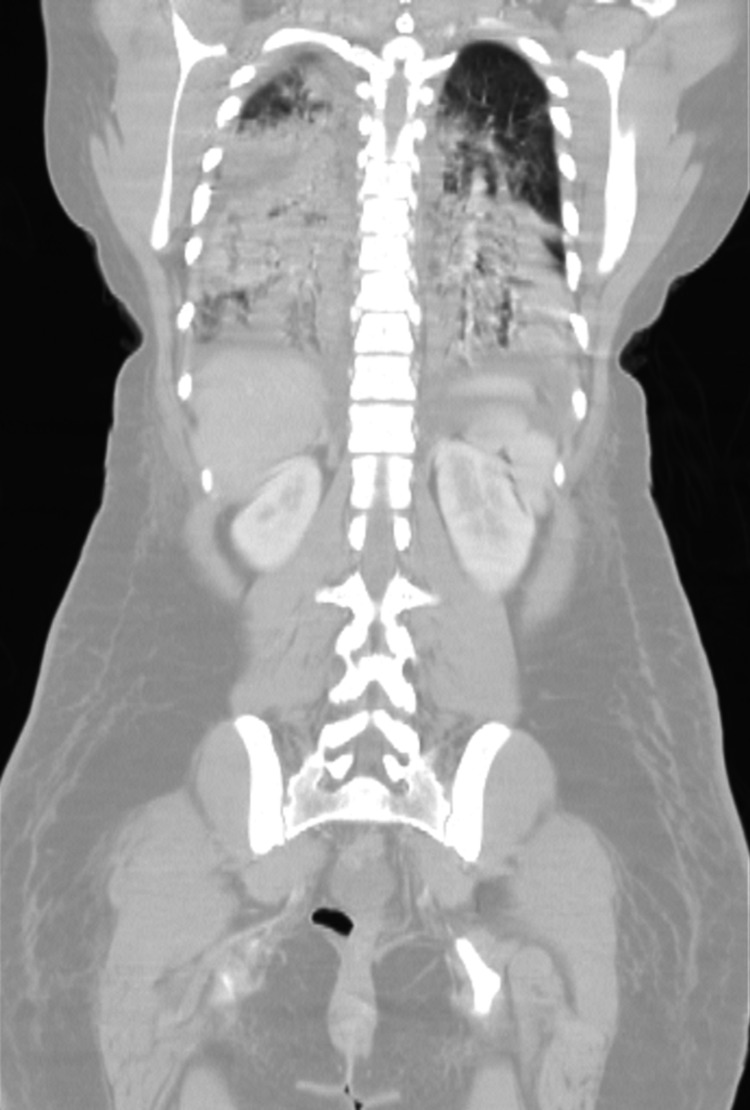
CT chest showing bilateral infiltrates from posterior coronal view

The next day, the patient demonstrated improvement with decreased lactate, improved kidney function, decreased fecal incontinence, and increased physical activity despite persistent hypotension that required increased pressor requirements and steroids. Later that evening, the patient reported chest pain. EKG showed diffuse ST elevations consistent with pericarditis. Cardiac enzymes were elevated from admission, as seen in Table 1. A bedside echocardiogram showed no signs of cardiomyopathy. Repeat chest X-ray was unchanged. The chest pain self-resolved after approximately four hours. About 1.5 hours later, the patient developed a fever of 102°F with a respiratory rate of 38 and oxygen saturation as low as 89% on room air. Rhonchi were auscultated along the right lobes with the left side sounding clear. Chest X-ray now showed bilateral opacities with a right-sided predominance (Figure 1). Her oxygen requirements increased, and she transitioned to continuous positive airway pressure support (PEEP 12 cmH_2_O, FiO_2_ 65%). Chest CT could not be performed immediately, thus empiric furosemide and full dose enoxaparin for possible fluid overload or pulmonary embolism (PE) were used. CT of the chest was completed early the following morning, which noted multifocal pneumonia with probable ARDS and no evidence of PE (Figure 2). Shortly after, the patient’s O_2_ saturation decreased to 78%. She was then intubated with initial ventilator settings of 14 PEEP and 100% FiO_2_. Arterial blood gas was notable for PaO_2_ of 52 mmHg (normal 80-100 mmHg). The inpatient team broadened antibiotics to cefepime, vancomycin, and doxycycline and arranged for air transport to a United States military hospital in Germany.

 

**Figure 3:**
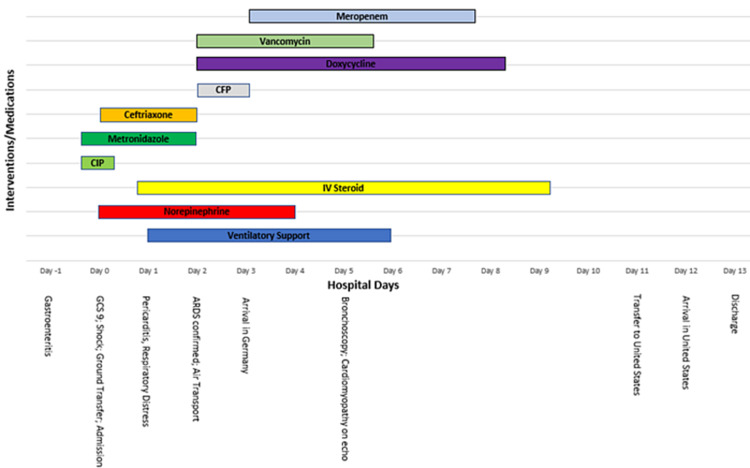
Timeline of events and treatment CIP: ciprofloxacin; CFP: cefepime; IV: intravenous: GCS: Glasgow Coma Scale; Echo: echocardiogram

While in Germany, cefepime was replaced with meropenem and the rest of her regimen antibiotic remained unchanged. The patient received bronchoscopy and bronchioalveolar lavage with no clinically significant findings on microscopy. She responded well to prone ventilation and was eventually extubated on day 6 without issue. Post transfer Troponin I was initially elevated at 12,316.2 ng/L (normal: 0-12) and peaked at 13,525.4 ng/L, with rapid decline thereafter. EKG was normal with a resolution of diffuse ST elevations. Vasopressors were stopped on day 4 and a repeat echocardiogram on day 5 showed mild left atrial enlargement, left ventricular systolic dysfunction, septal wall akinesis, and reduced ejection fraction at 46%. These findings were attributed to presumed myocarditis and further evaluation was deferred as the patient was hemodynamically stable at that time. Supportive treatment for myocarditis with beta-blockers and angiotensin-converting enzyme inhibitors was withheld given low baseline pressures in the 90s/60s mmHg when off vasopressors. All antibiotics were stopped on day 8 and by day 11 she was well enough for transfer back to the United States where she was discharged on day 13. The timeline of events and interventions are detailed in Figure 3.

Her broad infectious disease work-up from her entire hospital course fully resulted after discharge with no growth from respiratory, blood, urine, and bronchoalveolar lavage cultures. PCR of stool and respiratory samples were negative for several common and rare bacterial and viral pathogens. Notably, her enterovirus antibody titers returned markedly elevated at 1:80 dilution (fresh infection suspected at > 1:20) and Coxsackie B1-6 antibody titers were elevated ranging from 1:8 to 1:16 dilutions (negative < 1:8). Recommendations for convalescent serology to further evaluate were not acted on. Cardiac MRI performed months later as an outpatient revealed edema along the intraventricular septum with diffuse persistent late gadolinium enhancement consistent with myocarditis injury pattern.

## Discussion

We presented a case of an otherwise healthy female who was treated for symptoms of gastroenteritis and returned a day later with a GCS of 9 and subsequently developed septic shock, myocarditis, and severe ARDS. Evaluation for inciting etiology was thorough with many bacterial, fungal, and viral causes of gastrointestinal and respiratory infections excluded with negative tests and cultures. Cardiac MRI showing a myocarditis injury pattern provides a strong clue to a potential pathogen. Coxsackie B is brought into consideration as it causes gastrointestinal distress and is one of the leading causes of viral myocarditis with PCR evidence of it found in up to 33% of endomyocardial biopsies (EMB) from myocarditis patients younger than 35 [4,5]. Additionally, Coxsackie B is associated with encephalitis, which may explain the decreased GCS but sepsis and hypovolemia secondary to diarrhea can explain this finding as well [5]. Evidence of antibodies against enterovirus and multiple Coxsackie B serotypes in our patient support the diagnosis but identifying specific serotypes is difficult given prior observations of a heterotopic effect of enteroviruses leading to amnestic antibody response toward other Coxsackie B strains [6]. Despite the lack of EMB or convalescent serologies that can confirm the diagnosis, we strongly suspect Coxsackie B played a role based on corroborating findings in this case. We found only one similar case in Japan where a reportedly healthy 44-year-old man was brought in unconscious and found to have ARDS secondary to suspected viral pneumonia; he later developed myocarditis with serology implicating Coxsackie B4 [7]. Our case differs as it initially started with gastrointestinal distress with no signs and symptoms of respiratory illness before the onset of ARDS, thus this may be the first case detailing such progression with suspected Coxsackie B infection. Considering Coxsackie B rarely progresses to ARDS, we suspect that multiple factors were involved. 

ARDS primarily occurs in those who are already hospitalized with an injury that increases the risk of development. The onset of ARDS typically occurs within 72 hours of the injury for most patients with others taking up to a week to develop [1,2]. Risk factors are broken down into direct and indirect injuries of the lung pleura. Types of direct injury include pneumonia, aspiration of gastric contents, pulmonary contusion, inhalation injury, and near-drowning; whereas indirect injuries include sepsis, non-thoracic trauma, pancreatitis, major burns, drug overdose, blood transfusion, pulmonary bypass, and reperfusion edema [2,3]. Our patient’s presentation satisfies the temporal criteria with the inciting injury likely sepsis secondary to suspected Coxsackie B infection. Her decreased GCS on the second presentation suggests an increased risk for an additional injury; aspiration leading to pneumonia or chemical pneumonitis from gastric fluid. Her predominantly right-sided distribution of lung infiltrates on imaging correlates with this possibility. However, there are no reports of witnessed aspiration events or seizures that increase the likelihood. Furthermore, the use of several antibiotics by the time bronchoscopy was performed limit the ability to confirm aspiration pneumonia, and studies assessing for pepsin to confirm aspiration pneumonitis were not done. Given the lack of supporting evidence, the possibility of aspiration is only speculative.

Earlier studies by Ferguson et al. demonstrated only 6.5% of those with risk factors develop ARDS [8]. Attempts to refine risk assessment have led to the development of the lung injury prediction score (LIPS). This system assigns points to predisposing conditions (e.g., pneumonia, shock, sepsis, aspiration, pneumonia, high-risk surgeries, trauma) and accounts for modifiers (e.g., alcohol abuse, obesity, smoking chemotherapy, diabetes, FiO_2_, respiratory rate, O_2_ saturation <95%, acidosis) to produce a score [9,10]. Gajic et al. determined a score of ≥4 assessed at the time of admission demonstrated sensitivity of 0.69, specificity of 0.78, positive predictive value (PPV) of 0.18, and negative predictive value (NPV) of 0.97 with higher scores positively correlated with increased frequency of ARDS as high as 36% at scores ≥ 8 [10]. Based on their findings, Gajic et al. recommended LIPS as a tool to determine enrollment in future studies and make low-risk clinical decisions [10]. Despite the modest PPV, we consider it reasonable to err on the side of caution when caring for those with high LIPS points given the high mortality of ARDS, provided interventions are low risk. Conversely, the high NPV can help reassure clinicians of a low likelihood of ARDS, which can help justify not taking additional measures. Given our practice in the military, the application of LIPS at medically austere pre-hospital settings is enticing, but it likely would not change care since most injuries that increase the risk of ARDS typically warrant transfer to hospitals regardless of predicted ARDS risk. Thus, we consider LIPS as a tool best used at the time of admission to alert clinicians to increased ARDS risk, which can help determine facility suitability, justify an early transfer, or prime resources necessary to evaluate and treat.

Our patient’s progression to ARDS was initially unexpected due to factors such as her age, lack of comorbidities, predominant gastrointestinal signs, lack of pulmonary symptoms, and initial chest imaging being unremarkable. By using pre-admission data to calculate LIPS, our patient is attributed 2 points for shock, and 1 point each for sepsis, hypoalbuminemia, and decreased O_2_ saturation for a score of 5. Her score maybe 1-3 points higher if additional labs assessing for acidosis were performed or if aspiration was observed. A score of 5 imparts about a 12% chance risk for developing ARDS based on observations by Gajic et al. [10]. We consider this high enough to warrant maintaining the readiness of medical resources required to intervene at first signs of ARDS. If her risk was identified, it may have led to earlier recognition of ARDS at the first sign of respiratory distress and allowed for resources to be readily available to assess severity through blood gases or rule out other conditions such as PE, which may have eliminated the need for empiric measures. Overall, the outcome of this case was favorable, and the decisions made were reasonable given the limitations. The lessons learned to highlight the value that early identification of high ARDS risk would bring to similar cases.

The ability to assess the risk of development of ARDS allows the study of preventive interventions. Using LIPS as a basis to assign study participants for potential interventions has been used in two trials so far. The first used aspirin as a means to intervene in the inflammatory cascade leading to ARDS, but showed no benefit compared to placebo [11]. Whereas, the use of inhaled budesonide and formoterol combo in a small multicenter randomized control trial showed improved lung function as measured by SpO_2_:FiO_2_ with no progression to ARDS in the treatment group compared to placebo [12]. Findings such as this warrant further study and increase the possibility that ARDS may one day be preventable.

## Conclusions

This was a case of suspected Coxsackie B infection in a healthy adult who presented with gastroenteritis and progressed to septic shock, myocarditis, and severe ARDS. The occurrence of ARDS was unexpected in this case, but the risk could have been assessed using LIPS. There are currently no treatments that can prevent ARDS; risk assessment at the time of admission can help clinicians determine appropriate levels of care.
